# Differences in the knowledge, attitudes, and needs of caregivers and healthcare providers regarding palliative care: a cross-sectional investigation in pediatric settings in China

**DOI:** 10.1186/s12912-024-02052-2

**Published:** 2024-06-06

**Authors:** Xi Lin, Guo Qulian, Yongqi Bai, Qin Liu

**Affiliations:** 1 Department of Pediatrics, Children Hematological Oncology and Birth Defects Laboratory, The Affiliated Hospital of Southwest Medical University, Luzhou, 646000 Sichuan P. R. China; 2 Sichuan Clinical Research Center for Birth Defects, Luzhou, 646000 Sichuan P. R. China; 3 Department of Nursing, The Affiliated Hospital of Southwest Medical University, Luzhou, 646000 Sichuan China

**Keywords:** Palliative Care, Pediatrics, Knowledge, Attitude

## Abstract

**Background:**

Countries abroad have implemented pediatric palliative treatment for several years; however, complete pediatric palliative treatment guidelines and legal guidance remain lacking in China, making the implementation of palliative care difficult.

**Purpose:**

This study aimed to understand the current situation, similarities, and differences in the knowledge, attitudes, and needs of caregivers and healthcare providers regarding palliative care for children.

**Methods:**

This cross-correlation study collected data from pediatric and neonatal intensive care units of four hospitals in China. The participants comprised 180 caregivers and 172 healthcare providers. The measurement tools included the palliative care knowledge scale, palliative care attitude scale, and pediatric palliative care needs scale. This study adhered to the STROBE reporting guidelines.

**Results:**

Significant differences in palliative care knowledge, attitudes, and needs were observed between caregivers and healthcare providers (*p* < 0.005). Pediatric healthcare providers demonstrated higher knowledge and positive attitudes regarding palliative care than caregivers. Needs for communication in general and relief from pain and other symptoms of caregivers were higher than those of healthcare providers (*p* < 0.001). Furthermore, palliative care attitude and needs of healthcare providers were positively correlated with each other (*r* = 0.212, *p* < 0.005).

**Conclusion:**

Pediatric healthcare providers and caregivers demonstrated different perceptions of needs for palliative care. The results of this study can serve as a reference for the future development of guidelines related to children’s needs and related interventions.

**Supplementary Information:**

The online version contains supplementary material available at 10.1186/s12912-024-02052-2.

## Introduction

The World Health Organization defines pediatric palliative care (PPC) as a care method that effectively improves the quality of life and solves the problems faced by children who are at a risk of death from life restriction diagnosis to the end of life, e.g., early identification, comprehensive evaluation, and planned intervention regarding the physiological, psychosocial, and mental problems of children and their families to alleviate their suffering [1]. Consensus among domestic and foreign experts has emphasized that improving child survival rate is one of the goals of medical care for children [2]. The focus of diagnosis and treatment, when the outcome of a child’s death cannot be avoided, should shift from active treatment interventions to soothing and peaceful care, providing comfortable care and managing symptoms to minimize the pain of the child, thereby improving their quality of life in the final stage and providing psychological, emotional, and other support to the child’s family to alleviate the sadness due to bereavement [3].

China has paid relatively low attention to PPC, leading to its high demand [4]. Moreover, the use of palliative care services for patients remains low, and a systematic understanding of the assessment content and tools required for palliative care needs is lacking [5, 6]. Compared with other countries, the practice of PPC in China is in its early stages; thus, service resources are insufficient, and the service model is relatively simple [7]. Preliminary exploration efforts began in Beijing, Shanghai, Chengdu, Changsha, and other cities, in addition to Hong Kong, Macao, and Taiwan [8]. In the absence of specialized institutions, inhospital PPC services are predominantly offered by children’s specialized hospitals or related departments. Some individual hospitals have established palliative medicine departments to provide PPC services; however, the vast majority of practitioners lack professional training or education in this field [9]. Notably, China has a limited number of specialized PPC services, such as Butterfly House, with the majority of the palliative care services being provided in hospitals [10].

The families of patients should have sufficient knowledge about palliative care to provide appropriate palliative care [11]. Only after gaining sufficient knowledge, they can understand the true meaning of palliative care and change their attitude toward it [12]. The attitude of patients toward information acquisition also affects the efficacy of doctor–patient information communication [13]. Studies have demonstrated that most palliative care interventions for children are considered toward the end of their life, more than within a month of death, and they continue to receive active treatment, resulting in delayed palliative care interventions [14]. The obstacles to palliative care intervention are mainly associated with caregivers and healthcare providers [15]. Caregivers may believe taboos and lack palliative care knowledge, whereas physicians may be afraid of harming the doctor–patient relationship and communication as well as the values of palliative care decision-making [2]. Conversely, nursing staff delay the timing of palliative care interventions due to the unwillingness of physicians and caregivers to recommend palliative care as well as due to unclear referral content and timing [16, 17].

Some studies [15, 18] have reported that caregivers are most concerned about the following aspects when experiencing the end of life in children: (1) relief of the child’s physical symptoms; (2) communication between caregivers and medical personnel; (3) support of caregivers’ decisions; and (4) healthcare providers’ care and other needs for caregivers. Research has revealed that healthcare professionals who are unable to provide parents with the need to take care of their child in the late stages can prolong the period of grief for parents after the child’s death [19]. Conversely, healthcare providers who take care of children at the end of life often feel pressure and face many difficulties, including (1) preparation for the child’s death, (2) communication pressure, (3) failure to reach a consensus between the medical team and parents on treatment goals, (4) inability to provide family support, and (5) control of symptoms to alleviate the pain experienced by the child [16, 17].

Therefore, caregivers and healthcare providers have different views on care when a child’s life is threatened or restricted due to illness, and healthcare providers are not very clear about the needs of caregivers [20]. A previous study reported that integrating the perspectives of patients, caregivers, and healthcare professionals at the end of life can improve the quality of care [21].

Healthcare providers and caregivers may perceive critically ill children differently [22]. Assuming similar knowledge, attitudes, and needs toward palliative care between two populations without considering potential differences can lead to overlooking crucial aspects of nursing [23]. Recognizing disparities in the concepts of palliative care between these populations can enhance our understanding of the nature and purpose of their practice. This understanding is beneficial for nursing, education, and research among healthcare providers and caregivers [24].

The disparities in knowledge and attitude between the two groups can be attributed to several factors. One factor is the societal division of labor and knowledge specialization, which creates disparities in individuals’ access to and receipt of information [25]. Another factor is that rigid consultation processes and dominant conversational dynamics hinder information sharing [26]. Previous studies have shown that both healthcare providers and caregivers concentrate on illness and medical treatments [3, 27]. In particular, caregivers assisting with palliative care also actively seek curative treatment options [28]. When children are not aware of their condition, caregivers constantly struggle between the need to tell and the guilt of not telling, causing great psychological stress [29]. Consequently, some caregivers desire supportive information from healthcare providers to leverage social support for families [30]. Caregivers’ desire for end-of-life information also signifies emergency preparedness. Research reveals unmet treatment-related knowledge and needs among pediatric patients’ families [7, 31]. Approximately 85% of communication time involves physicians providing disease and treatment information, neglecting the variation in patients’ knowledge preferences, attitudes, and needs across illness stages [32, 33]. The alignment of physicians’ perceptions with patients’ actual preferences is only approximately 45% [34]. In addition, time constraints and patient volume often lead physicians to focus on treatment-related information, sidelining social and psychological concerns [35]. This leads patients to believe that communication should focus solely on treatment-related information, excluding nontherapeutic issues [27]. However, this does not imply that patients do not require information on other aspects.

At present, studies on PPC in China are limited, and no studies have compared the knowledge, attitudes, and care needs of healthcare providers and caregivers regarding PPC. Moreover, a complete system for PPC remains unavailable, resulting in the lack of knowledge of healthcare providers on intervening in child care [7, 36]. Therefore, this study aimed to understand the differences and correlations in the knowledge, attitudes, and needs of caregivers and healthcare providers regarding PPC to provide a reference for the future development of local care courses and intervention measures. The results of this study will guide clinical healthcare providers to understand the caregivers of children at palliative care centers and improve their quality of care for such children.

## Methods

### Study design and settings

This study collected data from patients in pediatric intensive care unit (PICU) and neonatal intensive care unit (NICU) at four tertiary and first-class hospitals in China from March to May 2022. The study participants included caregivers, physicians, and nurses. All respondents provided informed consent and voluntarily participated in this study.

### Participants

#### Caregivers

This study used convenience sampling to select caregivers of children of caregivers who were being treated in the PICU or NICU of four hospitals in China. This study collected data from 180 individuals.

The inclusion criteria are children who are currently in the PICU or NICU as caregivers, are able to cooperate with verbal communication and character recognition skills, and are over 20 years of age to voluntarily complete a consent form. Exclusion criteria include language and communication barriers.

#### Pediatric healthcare providers

This study included 89 doctors and 83 nurses working in the PICU or NICU of four hospitals in China. Convenience sampling method was employed, and the participants were administered a questionnaire survey.

The inclusion criteria were doctors, nursing staff formally employed by PICU or NICU, and adults over the age of 20 years who voluntarily completed the consent form. Exclusion criteria included healthcare providers who have not cared for terminally ill patients and do not want to continue participating in the study for any reason.

### Sample size

The G-Power 3.1 statistical software was used to estimate the number of samples. Two tails were set (effect size = 0.3, power = 0.95, and α value = 0.05). In total, 134 samples were analyzed, and after considering the possibility of dropout or loss, 10% of the samples were added. The study participants were divided in two groups, with 150 individuals in each group. This study collected 197 questionnaires from caregivers and 189 questionnaires from healthcare providers. Incomplete and invalid questionnaires were excluded. Finally, valid questionnaires from 180 caregivers and 172 healthcare providers were included.

### Measures

The researchers contacted the unit heads of the PICU and NICU; informed them about the purpose, conditions, and participants of the questionnaire and the process of the study; and obtained the participants’ verbal consent. The participants were led to the conference room before starting to fill the questionnaire, and they were asked to complete the questionnaire in an undisturbed environment. The time provided to fill the questionnaire was approximately 10 min.

Caregiver sociodemographic variables included age, sex, relationship with children, marital status, religious beliefs, education level, financial resources, and palliative care knowledge.

The sociodemographic variables collected from healthcare providers included age, sex, education level, work unit, clinical experience, pediatric experience, religious beliefs, and hospice-related course attendance.

Data were collected using two versions of the sociodemographic questionnaire, one for caregivers and one for pediatric healthcare providers. The other three parts of the questionnaire were the same: (a) parental PELICAN questionnaire (PaPEQu); (b) palliative care knowledge scale; and (c) palliative care attitude scale.

### PaPEQu

A questionnaire developed by Lin [37] to assess parental experiences and needs during their child’s palliative care, PaPEQu, was used. This study evaluated the content validity of PaPEQu answered by 10 pediatric and child health experts, with a Cronbach’s alpha value of 0.85–0.89 and a content validity index of 0.82–0.91. The item-level face validity index (I-FVI) was higher than 0.85. The questionnaire items were divided into support of the family unit, communication in general, shared decision-making, relief from pain and other symptoms, continuity and coordination of care, and bereavement support. The questionnaire consisted of 30 questions, and each question was rated on a 5-point Likert scale. The higher the score, the more important the need is.

The authors contacted Zimmermann via email to obtain authorization and conduct localized debugging. Three experts with bilingual master backgrounds were invited to translate the questionnaire from English to Chinese and another three to translate from Chinese to English. Five nursing experts were invited to review the expert validity of the scale for the translated questionnaire.

### Palliative care knowledge scale

The palliative care knowledge scale compiled by Lin [38] was used to understand the concept and cognition of palliative care provided by caregivers and healthcare providers. In total, 12 questions were scored using a 2-point scoring method. Correct answer was given 1 point, whereas wrong or unclear answer was given 0 point. Questions 5 and 7 were scored in reverse. The higher the score, the more knowledge of palliative care. The content validity index was 0.89–0.93, and the questionnaire reliability Cronbach’s alpha was 0.856. I-FVI was 0.79–0.88.

### Palliative care attitude scale

This study used the palliative care attitude scale prepared by Lin [38], which consisted of eight items. Each item was scored on the 5-point Likert scale. Three, four, and seven items were scored in reverse. The higher the score, the more positive the attitude toward palliative care. The researchers obtained the consent of the authors for the scale and revised the subject part of each question following the original scale. The expert validity index was 0.90–0.94, and the questionnaire reliability Cronbach’s alpha was 0.862. I-FVI was 0.73–0.93.

### Data analysis

Epidata 3.1 software was used for data entry, and Statistical Package for the Social Sciences version 25.0 software was used for data analysis. Corresponding statistical methods were selected following different data characteristics. Descriptive statistics were used to analyze the general data, palliative care knowledge, attitudes, and care needs of the study participants. Mean (M), standard deviation (SD), and t-test were used to analyze the differences in the PPC knowledge, attitudes, and needs of caregivers and healthcare providers. Correlations among caregivers’ and healthcare providers’ attitudes and care needs in PPC were analyzed using Pearson’s correlation.

## Results

In total, 406 questionnaires were administered, but only 363 questionnaires were returned, with a survey completion rate of 89.4%. After excluding incomplete and invalid questionnaires, 352 (95.3%) questionnaires were finally analyzed.

### Sociodemographic and work characteristics of participants

The general characteristics of the caregivers are presented in Table 1. The caregivers were mostly mothers (112, 51.9%) and fathers (56, 31.1%), followed by grandfathers (12, 6.6%) and grandmothers (12, 6.6%). In addition, 86.7% of the caregivers had no religious belief, 65% had a bachelor’s degree, and 27.8% knew about palliative care, with their palliative care information sources mostly being healthcare providers (46%) and television broadcast (26%).

The general characteristics of the healthcare providers are presented in Table 1. The average age of the healthcare providers was 30.7 ± 3.88 years, of which 89 (51.7%) were physicians, 83 (48.3%) were nurses, and 87 (50.6%) had master’s degrees in education. Their working experience was 11.6 ± 7.7 years.

Background characteristics did not differ significantly between the caregivers and healthcare providers, except for years of education, which was significantly higher among healthcare providers.

**Table 1:** General characteristics of the study participants (*n* = 352)

Variable	Caregivers *N* = 180	Healthcare providers *N* = 172
	*N* (%)	
Sex		
Women	123(68.3)	133(77.3)
Men	57(31.7)	39(22.7)
Religious Belief		
Buddhism	8(4.4)	18(10.5)
Taoism	5(2.8)	6(3.5)
Other	11(6.1)	3(1.7)
None	156(86.7)	145(84.3)
Ward		
PICU	99(55)	101(58.7)
NICU	81(45)	71(41.3)
Educational background		
Associate (College)	57(31.7)	17(9.9)
Bachelors (University)	117(65)	68(39.5)
Masters	6(3.3)	87(50.6)
Do you know about palliative care?		
Yes	50(27.8)	126(73.3)
No	130(72.2)	46(26.7)
	**M (SD)**	
Age (years)	30.79(3.83)	37.45(8.87)

N = number of participants, %=percent of participants, M = mean, SD = standard deviation

### Comparison of knowledge and attitudes of caregivers and healthcare providers in PPC

As presented in Table 2, healthcare providers scored higher than caregivers in terms of knowledge about palliative care. The average knowledge level of healthcare providers (M = 4.23) was significantly higher than that of caregivers (M = 3.97) (*p* < 0.01). The three items with the lowest knowledge scores among caregivers and healthcare providers were as follows: “When a doctor implements palliative care for children with serious illness, he should inform the patient or his family about the treatment method”; “According to the needs of family members, palliative care will supplement the patient with sufficient nutrition and water, which can effectively increase the patient’s physical strength and reduce hunger”; and “Palliative care wards are more expensive than regular wards.”

**Table 2:** Palliative care knowledge score

Items	Caregivers (*N* = 180)	Healthcare providers (*N* = 172)		
	M(SD)	M(SD)	t	*p*
1. The primary objective of palliative care is to proactively enhance the quality of life for patients nearing the end of their lives, ensuring a dignified conclusion.	0.97(0.18)	1.00(0.00)	-2.42	0.016
2.Palliative care prioritizes respecting the patient’s autonomy by seeking consent prior to conducting any examinations or treatments, and ensures open communication with both the patient and their family members.	0.90(0.30)	1.00(0.00)	-4.35	0.00
3.Appropriate palliative care interventions can alleviate most pain and discomfort experienced by terminally ill patients.	0.79(0.43)	0.94(0.25)	-3.98	0.00
4.Palliative care proactively administers the most suitable treatments to patients.	0.73(0.45)	0.92(0.27)	-4.59	0.00
5.Palliative care equals euthanasia.	0.72(0.44)	0.92(0.27)	-4.92	0.00
6.Palliative care wards typically incur higher costs than regular wards.	0.02(0.12)	0.55(0.49)	-13.77	0.00
7.The palliative care ward is often perceived as a place where patients await death after ceasing active treatment.	0.72(0.44)	0.93(0.25)	-5.30	0.00
8.Palliative care encompasses both the patient and their family members.	0.63(0.48)	1.00(0.00)	-9.95	0.00
9.Palliative care is dedicated to providing holistic care to terminally ill patients, addressing their physical, psychological, and spiritual needs.	0.84(0.36)	1.00(0.00)	-5.73	0.00
10.According to the needs of family members, palliative care will provide the patient with adequate nutrition and hydration, effectively enhancing the patient’s physical strength and alleviating hunger.	0.07(0.25)	0.74(0.44)	-17.68	0.00
11.When implementing palliative care for terminally ill patients, physicians should inform either the patient or their family about the treatment methods.	0.13(0.34)	0.81(0.39)	-17.447	0.00
12.Palliative care teams comprise physicians, nurses, social workers, psychologists, and chaplains.	0.87(0.34)	1.00(0.00)	-5.129	0.00
Total score	0.61(0.11)	0.90(0.08)	-26.02	0.00

N = number of participants, M = mean, SD = standard deviation, t = test statistic, p = probability

**Table 3:** Palliative care attitude score

Items	Caregivers (*N* = 180)	Healthcare providers(*N* = 172)		
	M(SD)	M(SD)	t	*p*
1.I believe that pediatric palliative care can enhance the quality of life at the end of life rather than prolonging suffering.	4.13(0.77)	4.53(0.60)	-5.41	0.00
2.I believe that palliative care for children can alleviate familial fear, anxiety, and sadness.	3.67(0.79)	4.24(0.75)	-6.90	0.00
3.I believe that palliative care leads to a perception of hopelessness regarding the future of the child’s life.	3.77(0.81)	4.83(0.38)	-15.53	0.00
4.I believe that receiving palliative care compels the child to confront the reality and pain associated with death.	3.20(1.22)	3.67(1.14)	-3.75	0.00
5.I believe that a child receiving palliative care may feel abandoned by family members or healthcare workers.	4.00(0.87)	4.28(0.58)	-3.56	0.00
6.I believe that if a child receives palliative care, it is to forgo or stop all treatment.	3.56(1.16)	4.68(0.46)	-11.79	0.00
7.Clinical healthcare providers are capable of administering terminal medical care in accordance with the advance directives of family members (legal representatives).	3.87(0.97)	3.76(0.61)	1.26	0.20
8.Receiving palliative care respects the child’s terminal medical wishes.	3.90(0.94)	4.17(0.64)	-3.09	0.00
Total score	3.76(0.36)	4.27(0.29)	-14.39	0.03

N = number of participants, M = mean, SD = standard deviation, t = test statistic, p = probability

Table 3 presents the difference between the mean values of attitudes between caregivers and healthcare providers. In terms of attitudes, a significant difference was observed between caregivers and healthcare providers (*p* = 0.03) (caregivers’ score, M = 3.76; healthcare providers’ score, M = 4.27). The item with the lowest attitude scores among caregivers and healthcare providers was as follows: “I feel that receiving palliative care will force the child to face the reality and pain of death.”

### Comparison of PPC needs between caregivers and healthcare providers

The results revealed that the overall PaPEQu mean score in the area of caregiver needs (*M* = 4.36, *SD* = 0.14) was higher than the overall mean score in the area of needs for healthcare providers (*M* = 4.14, *SD* = 0.28). On the subscales, caregivers scored higher than healthcare providers with respect to communication in general and relief from pain and other symptoms. The mean of communication in general was significantly (*p* < 0.001) higher for caregivers (*M* = 4.72 ± 0.22) than for healthcare providers (*M* = 4.40 ± 0.44) (Table 4).

**Table 4:** Scores of PaPEQu between caregivers and healthcare providers (*N* = 352)

Items	Caregivers (*N* = 180)	Healthcare providers(*N* = 172)		
	M(SD)	M(SD)	t	*p*
**Support of the family unit**	4.43(0.30)	4.30(0.45)	-0.12	-0.89
1.To have a place to sleep in the hospital close to my child.	4.80(0.40)	4.45(0.60)	6.46	0.00
2. To be involved my child’s care.	4.83(0.37)	4.50(0.66)	5.73	0.00
3. To have room where my family and I could spend some private time together.	4.33(0.79)	4.37(0.83)	-0.44	0.65
4. To have respite from the care of my child.	4.03(0.84)	4.44(0.72)	-4.86	0.00
5. To share my fears and worries with someone from the healthcare team.	4.20(0.78)	4.48(0.74)	-3.40	0.00
**Communication in general**	4.72(0.22)	4.40(0.44)	-8.40	0.00
6. To have the opportunity to ask questions at all times.	4.70(0.48)	4.65(0.54)	0.88	0.37
7. To find out how my child would die.	4.93(0.26)	4.57(0.61)	7.19	0.00
8. Let me know sooner the baby is dying.	4.63(0.61)	4.45(0.76)	2.43	0.01
9. To be informed early about my child’s imminent death.	4.90(0.30)	4.52(0.68)	6.81	0.00
10. To be supported in maintaining hope despite the hopeless.	4.47(0.72)	4.40(0.73)	0.92	0.35
**Shared decision-making**	4.57(0.27)	4.56(0.38)	0.69	0.95
11. To be involved in taking decisions.	4.60(0.63)	4.53(0.70)	0.91	0.36
12. That my personal beliefs and were considered when taking Decisions.	4.13(0.78)	4.58(0.65)	-5.86	0.00
13. Not to have the feeling that I had to take decisions all by myself.	4.50(0.58)	4.53(0.66)	-0.43	0.66
14. That the cessation of life-sustaining measures was Discussed with me.	4.87(0.34)	4.59(0.61)	5.33	0.00
15. That the measure to resuscitate my child were discussed with me.	4.77(0.47)	4.62(0.54)	2.76	0.01
**Relief of pain and other symptoms**	4.29(0.27)	3.86(0.39)	12.25	0.000
16. That my child received enough medication to ease her/his suffering.	4.96(0.20)	4.67(0.56)	6.40	0.00
17. That my child was awake and reseptive enough to be able to play/speak/or do things with us or other people around.	4.83(0.43)	4.28(0.76)	8.26	0.00
18.That my child received medication to calm.	3.93(0.65)	3.73(0.85)	2.48	0.01
19. That my child received complementary and alternative Medicine.	4.27(0.75)	3.84(0.78)	5.21	0.00
20. To be able to use nonpharmacological measures to ease my child’s suffering e.g.massage.	4.63(0.51)	4.14(0.91)	6.20	0.00
21. That my child received fluids until the end.	3.17(1.13)	2.51(0.70)	6.54	0.00
**Continuity and coordination of care**	4.34(0.43)	4.01(0.49)	6.71	0.27
22. To have a professional from the healthcare team to coordinate the care of my child.	4.57(0.61)	4.36(0.59)	3.20	0.00
23.To have the same physician providing care.	4.50(0.65)	4.03(0.95)	5.40	0.00
24. That my child’s care was mostly provided by the same nurses.	3.97(0.79)	3.65(0.80)	3.75	0.00
**Bereavement support**	3.91(0.28)	3.84(0.41)	1.80	0.71
25. To have the choice of where child might die.	4.43(0.68)	4.26(0.92)	2.05	0.04
26. That family and friends could say goodbye to my child.	4.53(0.57)	4.50(0.69)	0.40	0.68
27. That I was supported by the healthcare team attended my child’s funeral or burial.	4.27(0.72)	4.27(0.81)	-0.01	0.99
28. To take my child home after her/his death so that family and friends could saygoodbye.	4.67(0.48)	4.38(0.68)	4.57	0.00
29. That someone from the healthcare team attended my child’s funeral or burial.	2.89(0.66)	2.95(0.72)	-0.71	0.47
30. To stay in contact with someone from the healthcare team after My child’s death.	2.70(0.69)	2.73(1.12)	-0.27	0.78
**Total score**	4.36(0.14)	4.14(0.28)	9.22	0.00

N = number of participants, M = mean, SD = standard deviation, t = test statistic, p = probability

### Correlation of knowledge, attitude, and need scores

A borderline significant difference was observed between caregivers and healthcare providers in terms of relief from pain and other symptoms (*p* < 0.001) (caregivers’ score: *M* = 4.29 ± 0.27; healthcare providers’ score: *M* = 3.86 ± 0.29) (Table 5).

Pearson correlation was used to analyze the results of healthcare providers’ knowledge, attitudes, and needs regarding palliative care, which revealed a significant positive correlation between healthcare providers’ attitudes and needs (*r* = 0.212, *P* = 0.03; Table 6).

**Table 5:** Correlation analysis results for knowledge, attitudes, and needs scores among caregivers (*n* = 180)

Variables	Knowledge	Attitude	Needs
Knowledge	1		
Attitude	0.111	1	
Needs	0.229	0.078	1

**Table 6:** Correlation analysis results for knowledge, attitudes, and needs scores among healthcare providers (*n* = 172)

Variables	Knowledge	Attitude	Needs
Knowledge	1		
Attitude	0.015	1	
Needs	0.053	0.212*	1

*p < 0.005: two-tailed, based on Pearson’s correlation analysis

## Discussion

The present study found that knowledge about palliative care among caregivers and healthcare providers still needs to be strengthened. The main difference is that communication and alleviation of pain and other symptoms are the most important care needs for caregivers when the child’s treatment is not as expected and they require palliative care.

The results of this study indicated that the families of children with terminal illness had a low overall score on palliative care knowledge and that the three items with the lowest scores among caregivers and healthcare providers were the same. Considering the influence of traditional culture and other social factors, the general population, such as the family members of the patient, was found to have a high level of misunderstanding of the concept of PPC [28, 39, 40]. Resistance to PPC services and unwillingness to receive palliative care information were also noted. This suggests the need to accelerate the popularization of the concept of PPC and improve society’s understanding of PPC [41].

The results of this study also indicated that healthcare providers had higher scores in palliative care knowledge, which may be due to the different research subjects [7]. The healthcare providers assigned in the PICU and NICU who were included in this study faced more severe pediatric conditions, handled more death situations, and had a higher reserve of palliative care knowledge than the general pediatric healthcare providers. However, some PICU and NICU healthcare providers were also found to have misunderstandings about palliative care knowledge, particularly in terms of fees, disease notification procedures, and treatment, which affected their willingness and enthusiasm to provide palliative care services.

This study revealed significant differences in attitudes toward palliative care between caregivers and healthcare providers. In China, Confucian culture is deeply ingrained in traditional culture, viewing death in terms of “good life” and “evil death” [42]. Over time, influenced by Confucianism, Taoism, Buddhism, superstition, and folk customs, a prevailing mindset has emerged, emphasizing the avoidance of death and valuing life over death [43]. Death is often perceived as ominous, with individuals tending to adopt an evasive and negative attitude toward it, especially in the context of a child’s death, leading parents to adopt an avoidance attitude [43, 44]. In contrast, healthcare providers exhibit a more positive attitude toward palliative care, which is related to their proficiency in palliative care knowledge. Indeed, increased knowledge correlates with a more positive attitude toward palliative care [7].

Diverse cultural perspectives on death exist among different countries. Western views, influenced by religion and a belief in the separation of the soul from the body, prioritize the soul over the body and embrace a rational understanding of death. In addition, the widespread development of life and death education in the West fosters a relatively calm acceptance of death, facilitating more direct doctor–patient communication [45, 46]. Many obstacles exist for PPC in China, including cultural barriers, which may not be openly expressed by patients or their families, leading to misinterpretation of medical information and palliative care choices [44, 47]. Multicultural nursing requires consideration of patients’ cultural backgrounds, religious beliefs, worldviews, and values to provide effective care. Therefore, cultivating cultural competence and religious literacy among healthcare providers is crucial [48]. Furthermore, strengthening cultural exchanges with foreign countries can contribute to improving caregivers’ attitudes toward palliative care.

This study found that caregivers have a higher demand for general communication, pain relief, and other symptoms than healthcare providers, which affects the choice and experience regarding palliative care services of families. Research has shown that the most common symptom of PICU is uncontrolled pain, with the incidence of moderate-to-severe pain ranging from 40–50% [49]. Children with malignant tumors have high pain scores and a high demand for symptom management and pain control. Pain management is a core component of palliative care. Based on the age and cognitive abilities of the patient, standardized pain assessment scales are selected, and proactive measures are taken to improve pain symptoms and emotional health [50]. Simultaneously, PPC requires complementary and collaborative professional abilities among multidisciplinary team members, such as pain management, wound care, and psychological and social needs according to their professional strengths [51]. Thus, it is important to establish a long-term growth plan for multidisciplinary teams, regularly conduct team training, share case studies, and discuss and reflect.

Furthermore, research has shown that more than half of PICU healthcare providers face communication problems with the families of patients with terminal illness [52, 53]. According to reports, family members of 50% of PICU patients cannot understand the doctor’s explanations about the diagnosis, treatment, and prognosis of the patient’s disease. When the patient’s condition rapidly deteriorates, the family members lack sufficient understanding of the disease and cannot accept the facts, leading to medical disputes [54, 55]. Most children with tumors and their families hope that doctors can truthfully inform them of their prognosis and final time of life. This will enable early communication with doctors about how the patients will spend the rest of their lives, the mode and location of death, and how their families will prepare and respond after their death [27, 56]. In medical decision-making communications, traditional Chinese medicine practitioners should proactively assess patients’ desired information and distinguish between the needs of patients and caregivers. Communication content should be tailored based on patients’ preferences and the importance of the information for decision-making, with adjustments to the sequence of communication and the allocation of different content. It is essential to simultaneously address the information needs of both patients and caregivers, ensure effective decision-making communication, and actively engage patients in the doctor–patient communication process [7, 9]. This approach boosts patients’ willingness and ability to engage in the decision-making process.

Due to differences in growth, career, and experiential wealth as well as differences in knowledge, attitudes, and needs regarding palliative care between the two groups, healthcare providers may have had some experience managing patients requiring palliative care, and caregivers may consider this issue for the first time. This requires personalized services for parents. Some patients with severe illnesses already consider palliative care issues such as death and medical decision-making, but due to clinical situations, information acquisition, and other reasons, they are unable to express their own demands [4, 35]. Or, some family members may not be able to understand the special needs such as death [54]. For families who cannot accept illness and palliative care situations, healthcare providers continue to provide routine treatment and care in the hospital based on family treatment requirements. Although medical and social workers do not mention words such as “peace” and “end of life,” they provide *de facto* palliative care and grief counseling to family members [27].

### Limitations

A total of 43 caregivers refused to fill out the form, mainly because they considered it taboo. This finding indicates bias in the data, implying that we may have missed out on more negative concepts about palliative care. Nevertheless, it is worth noting that the cultural commentary is that these topics are so taboo that they even hinder survey completion.

This was a cross-sectional study, which only represented the views of caregivers and healthcare providers at a specific time and cannot infer causality. In addition, these tools, knowledge, and attitudes are specifically designed for laypeople, offering relatively simple questions; therefore, the knowledge and attitude scores of healthcare professionals are higher, and the knowledge score results exhibit a certain degree of bias.

## Conclusion

This study indicated that there is still room for improvement in the palliative care knowledge of caregivers and healthcare providers, valuing the similarities and differences in their needs and providing psychological counseling and necessary support. Based on the recognition that different populations share knowledge, attitudes, needs, and other aspects related to palliative care, interdisciplinary collaboration can be improved. When discussing death issues with affected children and their families, one should be culturally sensitive, pay attention to understanding their cultural characteristics, use appropriate communication methods, and gently ask them to understand and respond to pain. We believe that we must cherish the similarities and differences in concepts, attitudes, and needs of palliative care, as they may add value to patient care and collaboration and may help improve patient care.

### Electronic supplementary material

Below is the link to the electronic supplementary material.

Supplementary Material 1

## Data Availability

Data is provided within the manuscript or supplementary information files.
